# A Rare Case of Cavernous Intramuscular Hemangioma of the Forearm

**DOI:** 10.7759/cureus.52118

**Published:** 2024-01-11

**Authors:** Taha El Aissaoui, Adnane Lachkar, Najib Abdeljaouad, Hicham Yacoubi

**Affiliations:** 1 Department of Trauma and Orthopaedics, Mohammed I University, Oujda, MAR; 2 Department of Traumatology and Orthopedics, Mohammed VI University Hospital, Oujda, MAR; 3 Faculty of Medicine and Pharmacy, Mohammed I University, Oujda, MAR

**Keywords:** tumor, total excision, forearm tumor, musculoskeletal tumor, intramuscular hemangioma

## Abstract

Cavernous intramuscular hemangiomas are a part of a group of rare benign tumors. This case report outlines a unique instance involving a 72-year-old patient who sought medical advice at our department due to a progressively enlarging mass in the posterior aspect of her right forearm. Physical examination revealed a painless, mobile mass with no apparent skin abnormalities. Radiographs showed normal results, and an MRI raised suspicion of a fibrous tumor. A subsequent biopsy confirmed the diagnosis of cavernous hemangioma. The patient underwent a total excision procedure, resulting in favorable outcomes with no observed functional impairment or tumor recurrence over four years. This case is notable for its singularity, involving both the advanced age of the patient and the uncommon location of the tumor.

## Introduction

Cavernous hemangiomas belong to a broad spectrum of benign vascular tumors commonly observed in infancy [1]. Liston, in 1843, was the first to document an intramuscular hemangioma and labeled it an "erectile tumor" [2]. Merely 8% of cases receive a diagnosis through clinical evaluation, emphasizing the indispensability of imaging modalities like ultrasound and CT scans, especially in the diagnostic process [3]. These imaging techniques provide valuable insights into the tumor's location, size, and vascular nature [4]. Biopsy samples subjected to histological analysis further contribute to confirming the diagnosis and comprehending the histopathological characteristics of intramuscular hemangiomas.

Surgical excision is the treatment of choice [5]. In the case of recurrent or invasive hemangiomas, embolization or radiation therapy can be considered as secondary therapeutic options [4]. The uniqueness of the case presented here lies in the atypical location of the hemangioma, i.e., the forearm, and the age of our patient (72 years).

## Case presentation

A 72-year-old female with a medical history of type 1 diabetes and a transtibial amputation done 14 years ago presented to our department due to a growing mass in the posterior aspect of the right forearm that developed over two years. The primary concern was the size of the mass; the patient did not report anorexia, weight loss, or asthenia.

**Figure 1:**
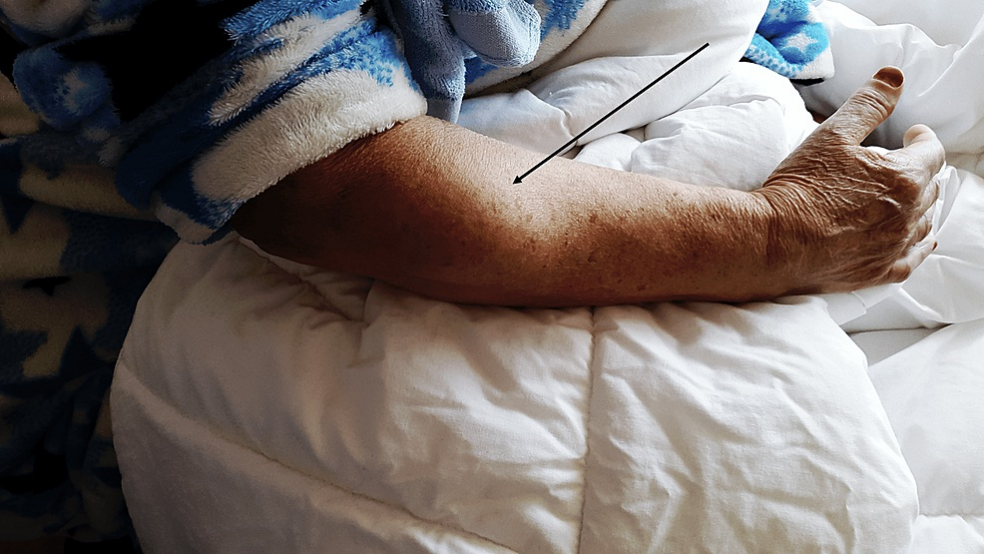
Clinical image showing a mass developed in the posterolateral aspect of the right forearm

The physical examination revealed a mass measuring 80 mm/40 mm in diameter on the posterior aspect of the right forearm. The mass was mobile, painless, and exhibited no skin lesions or abnormalities. The shoulder, elbow, and hand examination revealed no limitation in the range of motion, and the neurovascular evaluation revealed no abnormalities. Axillary lymph node evaluation yielded normal results (Figure 1).

**Figure 2:**
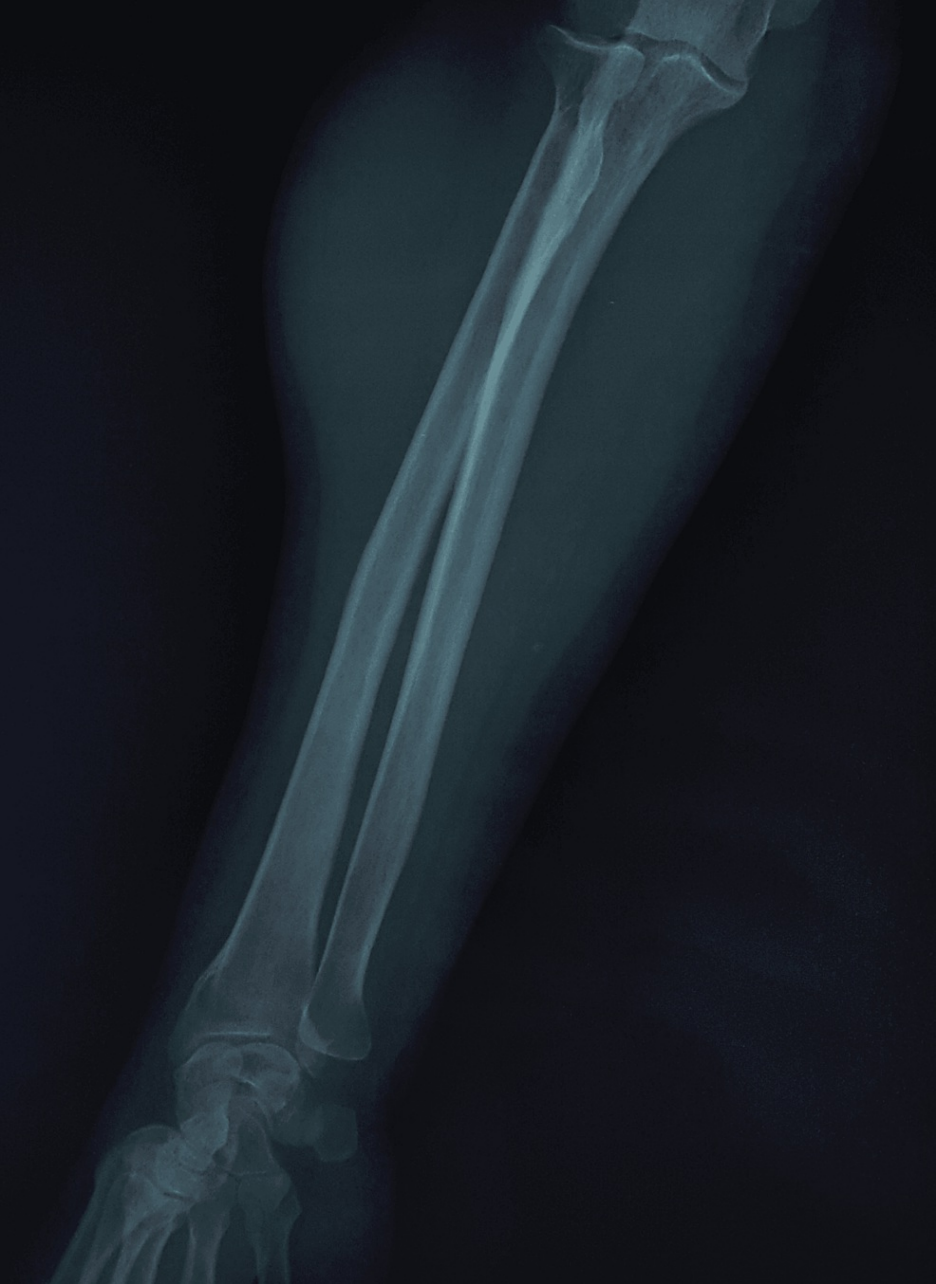
Anteroposterior X-ray view of the right forearm

**Figure 3:**
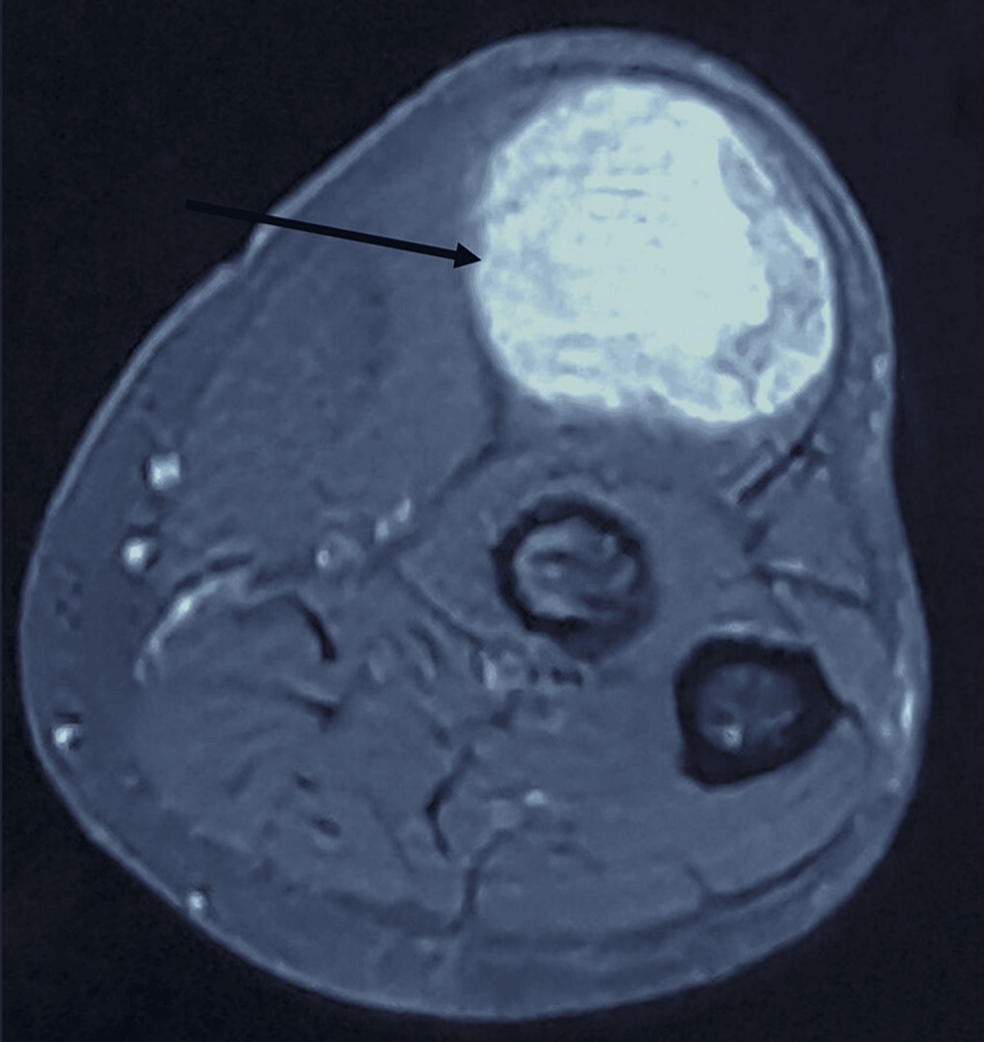
Axial proton density fat-saturated MRI view of the right forearm revealing a mass in the right forearm's posterolateral aspect

**Figure 4:**
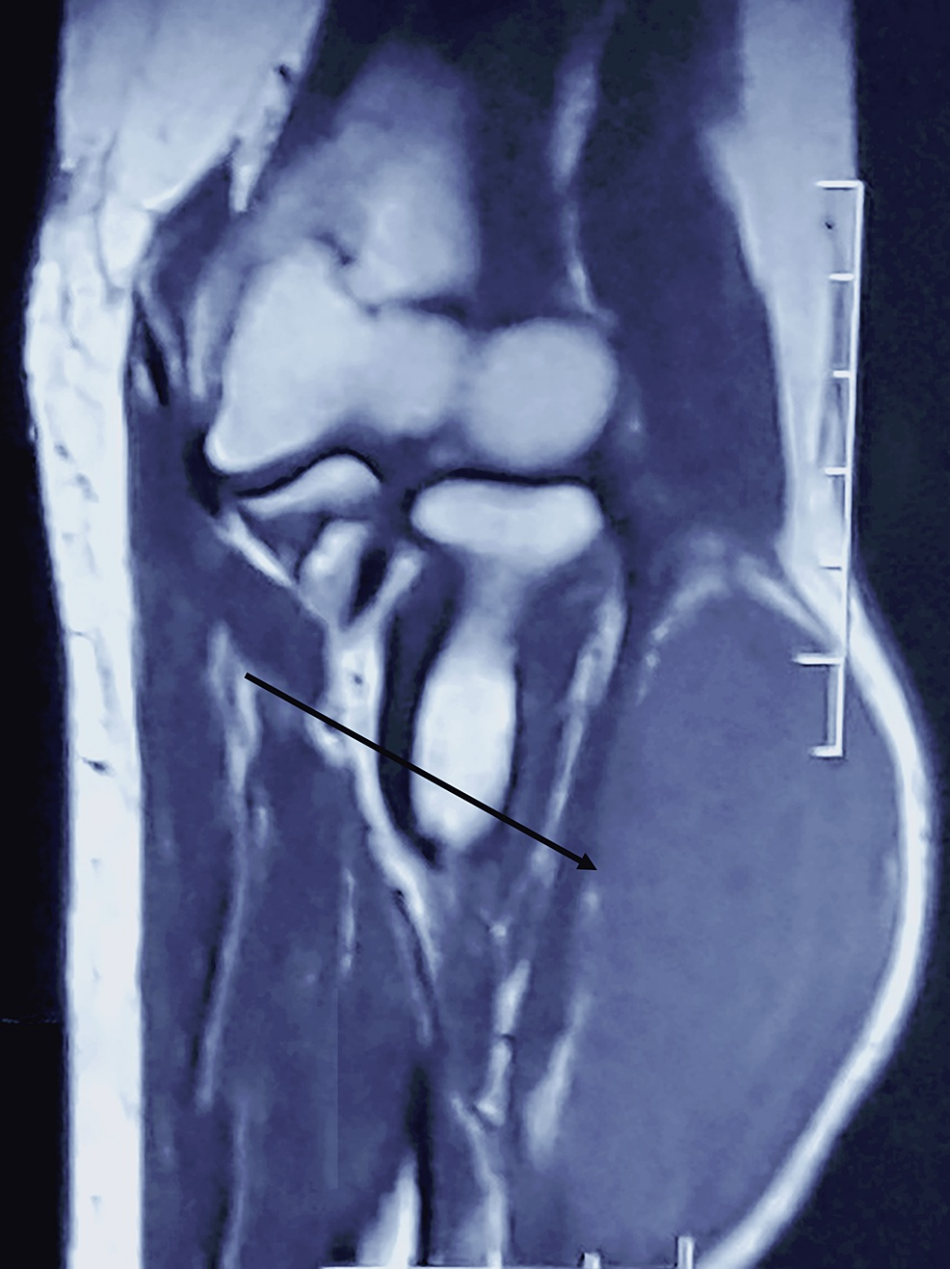
Sagittal T1 MRI view of the right forearm

**Figure 5:**
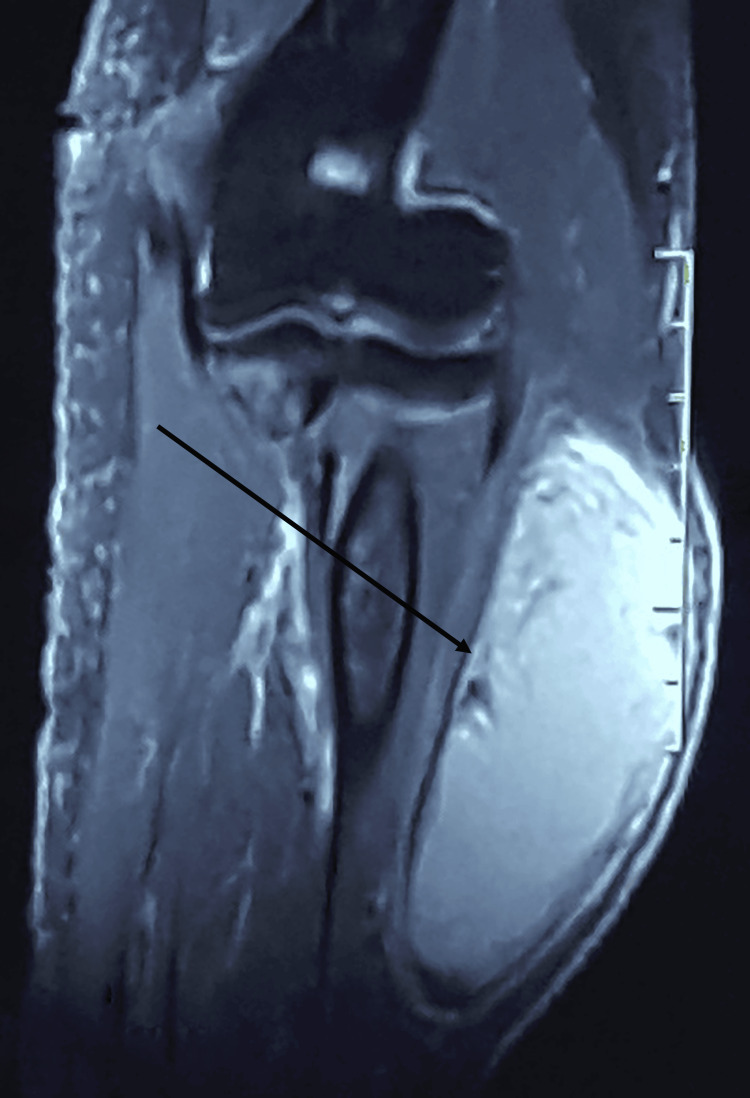
Sagittal T1 gadolinium MRI view of the right forearm

X-rays showed no calcifications or bone abnormalities (Figure 2). An MRI of the right forearm revealed a hypervascularized, well-defined mass measuring 37 mm/34 mm/79 mm, situated in the joint extensor muscle. The mass exerted pressure on the radial carpal extensor muscle, with no bone abnormalities noted, suggesting a solitary fibrous tumor (Figures 3-5).

**Figure 6:**
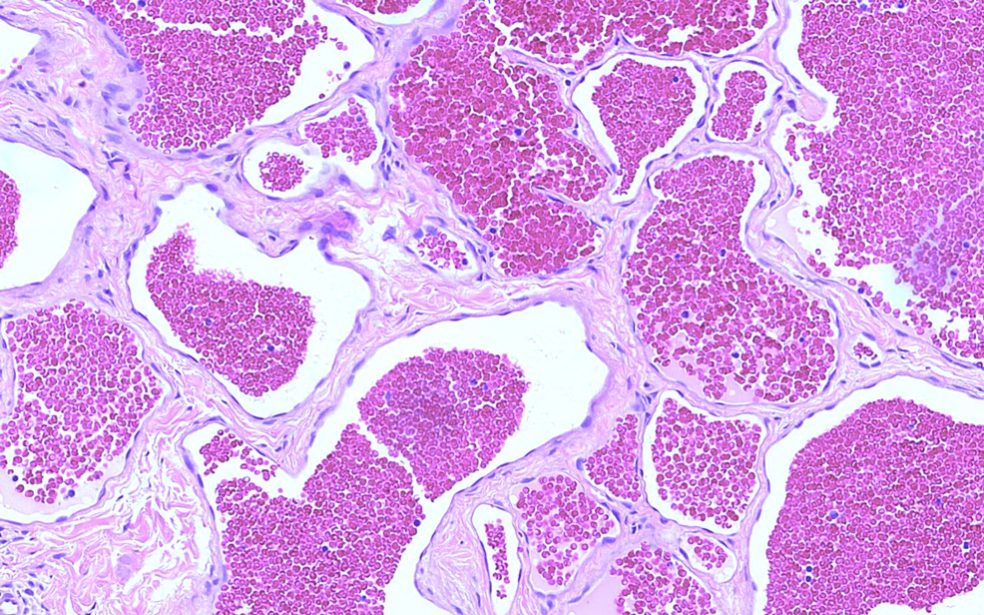
Histopathologic image of the biopsy A photomicrograph of the lesion shows clusters of cavernous spaces filled with blood, separated by connective tissue septa. The endothelial cells lining the blood vessels are flattened with no evident nuclear atypia (H&E, x200).

**Figure 7:**
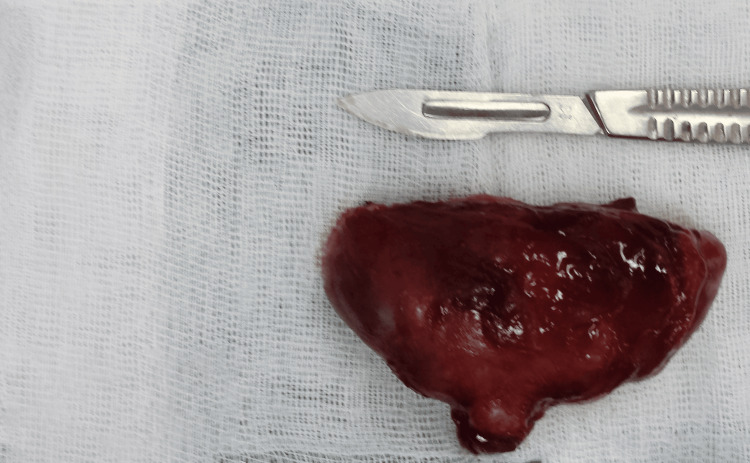
The macroscopic aspect of the tumor after excision The brownish color reflects the vascular structure of the tumor.

Subsequently, we performed a biopsy, leading to the diagnosis of the cavernous hemangioma (Figure 6). In a second procedure, the tumor was entirely resected (Figure 7).

Over a follow-up of four years, the patient exhibited positive outcomes with no functional impairment, complications, or tumor recurrence.

## Discussion

According to the literature, hemangiomas are classified into four types based on the predominant vascular channel: capillary hemangiomas, cavernous hemangiomas, arteriovenous hemangiomas and venous hemangiomas [6]. Cavernous hemangiomas are characterized by caverns lined with a single layer of endothelial cells, filled with large blood spaces, and separated by fibrous tissue [6]. They predominantly develop at the cutaneous, subcutaneous, and mucosal tissue levels, with intramuscular occurrences representing only 0.8% of diagnosed cases [7,8]. While rare, malignant transformation is possible [9]. Although these tumors can appear in any skeletal muscle, about half are situated in the lower extremities, with the thigh being the most frequent site [10]. Uncommonly, they may occur in the muscles of the upper limb, particularly, the elbow. Diagnosis typically occurs during young adulthood, affecting individuals under 30 in 80%-90% of cases and showing no sex preference [11]. Our case is unique regarding the age of occurrence and the tumor's location.

The tumor size influences clinical presentations of intramuscular hemangiomas, potentially leading to persistent pain, swelling, increased local temperature, discoloration of the overlying skin, and structural complications. Additionally, it can be a source of functional disability due to compression of the surrounding anatomical structures. Although rare, acute compartment syndrome can also occur [12].

The significance of imaging lies in identifying both vascular and nonvascular components of hemangiomas. Radiographs reveal calcifications and bone abnormalities such as periosteal reaction, cortical erosion, reactive sclerosis, coarsening of the trabeculae, and bony overgrowth. In 20% of intramuscular hemangiomas, characteristic calcifications known as phleboliths may be present [13].

MRI remains the gold standard for diagnosis, describing the tumor, location, size, and interaction with the surrounding anatomical structures and helping distinguish it from other tumors [14]. Despite these benefits, histopathologic confirmation remains mandatory for a conclusive diagnosis.

According to the literature, the therapeutic arsenal includes various options. Uslu et al. documented the reduction in size and symptoms of intramuscular hemangiomas through sclerotherapy [15]. Picci et al. proposed embolization as a potential alternative, particularly in extensively invasive infiltrating hemangiomas with challenging surgical excision [16]. Ultimately, surgery remains the preferred treatment in symptomatic cases, allowing for histopathologic examination and preventing tumor recurrence. It is crucial to emphasize that complete resection is imperative to minimize recurrence, with rates ranging widely from 9% to 28% [5]. In our case, surgical excision yielded positive results, with no recurrence of the tumor or functional impairment.

## Conclusions

This case report highlights the rarity of cavernous intramuscular hemangioma of the forearm, as seen in our elderly patient. The successful diagnosis, achieved through imaging, histopathology, and complete surgical excision, resulted in positive outcomes and a recurrence-free follow-up. Beyond its immediate clinical impact, this case contributes to the growing knowledge of diagnostic and therapeutic approaches toward such rare hemangiomas. While underscoring the importance of considering hemangiomas in the differentials of soft tissue masses, particularly in unusual demographics, the study acknowledges the necessity of continued vigilance due to the potential risk of recurrence. Regular follow-up protocols are recommended to ensure sustained positive outcomes and to inform future research endeavors in this domain.
